# Sickle cell disease prevention: How prepared are the senior secondary school students in Surulere Local Government Area, Lagos, Nigeria?

**DOI:** 10.4102/phcfm.v14i1.3260

**Published:** 2022-04-19

**Authors:** Oluchi J. Kanma-Okafor, Adetola O. Abolarinwa, Omobola Y. Ojo, Ekanem E. Ekanem

**Affiliations:** 1 Department of Community Health and Primary Care, Faculty of Clinical Sciences, College of Medicine, University of Lagos, Lagos, Nigeria; 2 Department of Community Medicine and Primary Care, Faculty of Public Health, Federal Medical Center Abeokuta, Abeokuta, Ogun State, Nigeria

**Keywords:** knowledge, attitude, sickle cell disease, genetic counselling, students, Lagos, Nigeria

## Abstract

**Background:**

Sickle cell disease (SCD), a common hereditary disease, can be prevented by preparing young people ahead of the conception of an affected foetus.

**Aim:**

To assess the knowledge and attitude regarding SCD amongst senior secondary school students in Surulere Local Government Area (LGA), Lagos, Nigeria.

**Setting:**

Senior secondary schools in Surulere LGA.

**Methods:**

This was a descriptive cross-sectional study amongst 300 senior secondary school students. Data were collected using a self-administered questionnaire and analysed using Stata16. The Chi-square and Fisher’s exact tests were used to determine the association between categorical variables. The level of significance was predetermined at *p* < 0.05.

**Results:**

The mean age of the respondents was 15.2 (±1.3) years, with a male-to-female ratio of about 1:2. The majority (90.0%) of the respondents were aware of SCD, 63.0% had good knowledge, although less than half of them (46.3%) knew SCD to be a blood disorder, whilst about two-thirds (53.1%) knew that it was an inherited condition. About one fifth (24.4%) of them knew about prevention by genetic counselling. The majority (97.0%) of them had a positive attitude towards SCD. Over two-thirds (72.6%) were aware of their genotype. The prevalence of SCD was 2.0%, whilst 18.9% of them were carriers of the sickle cell trait. Knowing their SCD status but not necessarily their genotype was significantly associated with their attitude towards the disease (*p* = 0.014).

**Conclusion:**

The prevention of SCD was not known to the majority, and better attitudes were more likely when the SCD status was known. Therefore, routine screening and counselling could potentially aid SCD control.

## Introduction

Sickle cell disease (SCD) is an autosomal-recessive genetically transmitted haemoglobinopathy that is responsible for considerable morbidity and mortality.^1^ Sickle cell disease results from a point mutation in the gene, a substitution of glutamic acid with valine at position six of the haemoglobin beta chain, causing an abnormality in haemoglobin synthesis, leading to the production of red blood cells that have an abnormal sickle shape.^2^ The different forms of SCD include sickle cell anaemia (haemoglobin SS [HbSS]), sickle cell haemoglobin C (Hb-SC) and sickle cell thalassaemia (Hb-SSthal).^2^

In Nigeria, the prevalence of HbSS is about 20 per 1000 births, meaning that in Nigeria alone, about 150 000 children are born annually with sickle cell anaemia.^3^ Children born to two parents with sickle cell trait have a 25% chance of having SCD and a 50% chance of having the sickle cell trait. Therefore, it is highly necessary for people of the reproductive age group to understand the genetics of SCD and know their blood type if they carry the S gene before selecting partners for future marriage.^4^

In SCD, the main pathology is the trapping of sickle-shaped red cells in small blood vessels that result in blockages and this usually manifests as bone pain, which is one of the most dreadful symptoms in people affected by SCD.^5^ This same process can result in other complications such as strokes, bone necrosis and kidney failure, and some affected persons are also likely to have stigmatising signs including jaundice, leg ulcers and short stature.^6^ Factors that enhance sickling include infection, dehydration, exhaustion, a change in temperature, et cetera.^7^

In addition, it is important to note that social and environmental factors are likely to contribute to the occurrence of psychopathology in SCD. A very important aspect of the social environment is the attitude and perception of non-sufferers towards affected persons.^8^ Sickle cell disease could be prevented if every conception of a child with SCD is avoided by assessing the risk of SCD based on prior knowledge of the genotype of the potential parents.^5^ Genotype awareness before a young person attains the time of the marriage decision is paramount to prevention of SCD, given that the disease is preventable.^5^ However, should a couple who both carry the sickle cell trait decide to proceed towards marriage, genetic counselling, which entails receiving information about how likely it is that genetic conditions like SCD might manifest in a family, proves a beneficial option towards limiting the risk of having a child with SCD.^9^ A couple is referred for genetic counselling particularly where the risk of SCD exists and the couple is planning for pregnancy.^9^ Genetic counselling proves to be a useful way to prevent SCD although some of the decisions taken after counselling may be devastating to the family.^9^

Senior secondary school students are likely to be in relationships that may eventually lead to marriage, so the issue of pre-marital screening is of importance but may be affected by the existing knowledge and attitudes to SCD.^10^ Therefore, understanding the inheritance pattern of SCD, its health significance, as well as the associated stigma towards individuals with SCD, particularly amongst secondary school students, is very important.^10^ This study assessed the knowledge and attitudes regarding SCD and its prevalence amongst secondary school students in Surulere Local Government Area (LGA), Lagos State, Nigeria.

## Methods

### Study design

This was a descriptive cross-sectional study.

### Study setting

Lagos State lies in the south-western part of Nigeria with a population of close to 20 million inhabitants and a population density of 47 111 inhabitants per square kilometre.^11^ This study was carried out in Surulere LGA, one out of 20 LGAs in Lagos State, which is divided into three Local Council Development Areas (LCDA), namely Surulere, Coker-Aguda and Itire-Ikate.^11^

### Study population

The study participants were senior secondary school students, attending schools in Surulere LGA. The senior students, who are usually much older than the juniors, are typically in their late teens and are, therefore, much likely to be deciding to get married and bear children soon.

### Sample size

The minimum sample size was determined using the Cochran formula (*n* = z^2^ × p × q/d^2^), with a standard normal deviate at 95% confidence interval of 1.96, a prevalence rate of 81.8% (proportion of students with adequate knowledge about SCD from a previous study)^12^ and the error of precision set at ± 5% (0.05). The minimum sample size, n, of 229 was calculated. To account for non-response, 20% of 229 was added to arrive at a sample size of 275. However, a total of 300 respondents were recruited for this study.

### Sampling strategy

A multistage sampling method was employed to select the respondents from the sampling population.

In the first stage, the simple random sampling method by ballot was used to select one LCDA out of three, whilst in stage two, six secondary schools from an average of about 14 secondary schools in each of the two LCDAs were selected by ballot. In the third stage, after proportionate sampling to determine the number of students to be selected from each of the six schools, from each school, in each of the three levels (SS1, SS2 and SS3), the systematic random sampling method was applied according to the classroom sitting arrangements of the students. The school register was used as a sampling frame, based on a sampling interval of one, therefore, every other student was selected for the study.

### Data collection

Data were collected using a pretested, semi-structured, self-administered questionnaire developed from a review of relevant literature.^10,13,14,15,16,17^ The questionnaire was divided into three sections: ‘Section A’ elicited the socio-demographic data of the respondents, ‘Section B’ assessed the knowledge of SCD, ‘Section C’ was designed to examine the attitude of secondary school students towards SCD, based on a 5-point Likert scale, whilst ‘Section D’ was designed to examine the respondents’ sickle cell status and their genotype, if known. The questionnaire was pretested amongst 30 senior secondary school students in a public secondary school in another LGA, which had a similar setting to the setting of the current study. After the pretest, the questionnaire was restructured to eliminate incomprehensible questions, to include constructs that adequately measured the required variables and to make instructions clearer.

### Data analysis

A scoring system^13^ was used to assess both the knowledge and attitude regarding SCD. In assessing knowledge, the raw score system was used. For every correct response, one point was awarded. For every incorrect response or non-response, a zero point was awarded. The total score for each respondent was converted to a percentage and graded as poor knowledge if it was < 50%, good knowledge if it is ≥ 50%. In assessing the attitude towards SCD, the points on the Likert scale were graded as five marks if the response was ‘strongly agree’, four marks if the response was ‘agree’, three marks if the response was ‘neutral’, two marks if the response was ‘disagree’ and one mark if the response was ‘strongly disagree’. The total attitude scores were then calculated for each respondent and graded as negative attitude if the score was below the median and positive if it is equal to or above the median. Data collected were analysed electronically using the Stata statistical software version 16. The results were presented in tables showing frequencies and proportions. The Chi-square and Fisher’s exact tests were used to test the associations between categorical variables. The level of significance was set at *p* < 0.05.

### Ethical considerations

Ethical approval for this study was obtained from the Health Research Ethics Committee (HREC) of the Lagos University Teaching Hospital (LUTH) (HREC assigned Number: ADM/DCST/HREC/APP/547). A letter of approval was granted by the authorities of the concerned educational district and permission was granted by the head of each of the schools before data collection was commenced. Written informed consent was obtained from each respondent before enrolment into the study after being informed of the study’s scope and objectives. Anonymity was maintained throughout the study. The respondents were assured of the confidentiality of their responses, and that the data collected would be used strictly for the study. Participation was purely voluntary and without coercion. The students were assured that there would be no consequences for non-participation.

## Results

Three hundred secondary school students participated in this study, with a response rate of 100%.

The mean age of the respondents was 15.2 ± 1.3 years, ranging from 10 years to 21 years. Most (88.7%) of the respondents were within the 14–17 years age group. There were more females (63.3%) than males (36.7%). Other socio-demographic characteristics are shown in Table 1.

**TABLE 1:** Socio-demographic characteristics of the respondents.

Variables	Frequency (*n* = 300)	Percentage (%)	Mean age ± s.d.
**Age group (years)**			15.2 ±1.3
10–13	22	7.3	-
14–17	266	88.7	-
18–21	12	4.0	-
**Gender**			
Female	190	63.3	-
Male	110	36.7	-
**Religion**			
Christianity	153	51.0	-
Islam	147	49.0	-
**Ethnic group**			
Yoruba	215	71.7	-
Igbo	61	20.3	-
Hausa	7	2.3	-
Others	17	5.7	-
**Mother’s highest level of education**			
No formal education	27	9.0	-
Primary	33	11.0	-
Secondary	136	45.3	-
Tertiary	104	34.7	-
**Father’s highest level of education**			
No formal education	22	7.4	-
Primary	22	7.4	-
Secondary	100	33.2	-
Tertiary	156	52.0	-
**Mother’s employment status**			
Employed	152	50.5	-
Unemployed	148	49.5	-
**Father’s employment status**			
Employed	219	72.9	-
Unemployed	81	27.1	-

s.d., standard deviation.

As shown in Table 2, the majority (90.0%) of the respondents were aware of SCD. Healthcare workers (25.8%) were the most frequently identified source of information, followed by friends and family (24.7%) and the internet (23.9%), but the mass media (television/radio [16.6%] and posters [9.0%]) were less recognised as sources of information on SCD. Out of those who had heard about SCD, 46.3% knew that it was a blood disorder, whilst 43.7% knew that SCD affects people of all ages. Inheritance from one’s parents was rightly the most frequently reported method of acquiring SCD, whilst about 21.8% wrongly stated that the disease was infectious. Over one third (70.0%) of the respondents identified frequent illness as a sign of SCD. The majority (81.9%) rightly identified blood testing as the means of diagnosing SCD. Regarding the risk of acquiring SCD per child if one parent had the sickle cell trait, respondents mostly identified it as a 50.0% risk, and only 4.8% of them rightly stated that there was no risk of SCD. The risk of acquiring SCD per child if both parents carried the sickle cell trait was mostly (56.3%) given as 100.0%, whilst only 4.1% correctly reported a 25.0% risk. The majority (70.0%) opined that the prevention of SCD was best achieved if the genotype was known well ahead of marriage.

**TABLE 2:** Respondents’ awareness, sources of information and knowledge of sickle cell disease.

Variables	Frequency	%
**Awareness (*n* = 300)**	270	90.0
**Sources of information† (*n* = 270)**		
Health professionals	123	25.8
Friends/Family	118	24.7
Internet	114	23.9
TV/Radio	79	16.6
Posters	43	9.0
**SCD is a blood disorder (*n* = 270)**	125	46.3
**SCD affects all age groups (*n* = 270**	118	43.7
**Mode of acquiring SCD † (*n* = 270)**		
Witchcraft/punishment from God	9	2.7
Inherited from parents	180	53.1
From an infected person	74	21.8
Insect bites	24	7.1
Direct body contact with a sufferer	27	8.0
Don’t know	25	7.3
**Sign/symptom of SCD known (*n*= 270)**		
Frequent illness	189	70.0
Yellow eyes	46	17.0
Don’t know	35	13.0
**How SCD is diagnosed (*n* = 270)**		
By blood test	221	81.9
By urine test	16	5.9
Don’t know	33	12.2
**The risk of SCD per child if one parent has the sickle cell trait (*n* = 270)**		
100%	17	6.3
50%	114	42.2
25%	64	23.7
Zero	13	4.8
Don’t know	62	23.0
**The risk of SCD per child if both parents have the sickle cell trait (*n* = 270)**		
100%	152	56.3
50%	41	15.2
25%	11	4.1
Zero	11	4.1
Don’t know	55	20.3
**Prevention of sickle cell disease †**		
Genetic counselling	66	24.4
Genotype testing long before being ready for marriage	189	70.0
Don’t know	15	5.6

SCD, sickle cell disease.

† , Multiple responses allowed; TV, television.

In Table 3, it is shown that 40.7% strongly agreed that SCD is not a spiritual problem and should not be ignored, whilst 37.8% believed that SCD is not the fault of one’s parents. However, the opinion that SCD could not be prevented by prayer and fasting was not popular. Whilst the greater proportion of the respondents agreed that SCD is a serious condition and that caring for a child with SCD would be very challenging, a smaller proportion believed that SCD could happen in any family. Regarding being aware of a partner’s sickle cell status, a larger proportion believed that it was important to know one’s genotype ahead of marriage. The ease of accessibility of testing for SCD in terms of cost was strongly expressed by 17.4%, whilst the majority (70.4%) supported a decision to test ahead of marriage.

**TABLE 3:** Respondents’ attitude towards sickle cell disease.

Statements (*n* = 270)	SA		A		N		D		SD	
	*n*	%	*n*	%	*n*	%	*n*	%	*n*	%
SCD is not a spiritual problem and should not be ignored.	110	40.7	81	30.0	31	11.5	30	11.1	18	6.7
SCD in a child is not the fault of one’s parents.	102	37.8	110	40.7	26	9.7	22	8.1	10	3.7
Couples cannot prevent SCD by fasting and prayer.	25	9.3	39	14.4	64	23.7	90	33.3	52	19.3
SCD is a serious condition.	113	41.9	119	44.1	21	7.8	10	3.7	7	2.5
Caring for a child with SCD would be very challenging.	113	41.9	105	38.9	28	10.4	18	6.6	6	2.2
SCD could happen in any family.	26	9.6	40	14.8	41	15.2	47	17.4	116	43.0
It is important to know whether one’s partner has the sickle cell trait.	125	46.3	119	44.1	12	4.4	8	3.0	6	2.2
It is important to know one’s genotype ahead of the marriage.	137	50.7	97	36.0	16	5.9	16	5.9	4	1.5
Testing for sickle cell trait is not difficult or expensive.	47	17.4	85	31.5	64	23.7	42	15.6	32	11.8
I would go for testing with my future partner before marriage.	190	70.4	67	24.8	10	3.7	2	0.7	1	0.4

SA, strongly agree; A, agree; N, neutral; D, disagree; SD, strongly disagree; SCD, sickle cell disease.

**FIGURE 1:**
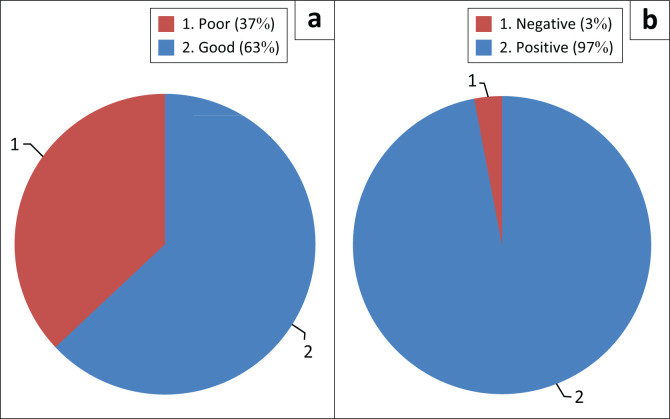
Respondents’ overall knowledge and attitude regarding sickle cell disease.

Overall, about 63.0% of the students had good knowledge about SCD, whilst the majority (97.0%) had a positive attitude regarding the condition (Figure 1). In Table 4, it is shown that over 70.0% of the students knew their sickle cell status, the majority (79.1%) having genotype AA, whilst about 18.9% had genotype AS. The SCD prevalence was found to be 2.0%. Of those who knew their genotype, 37.2% of them got tested out of curiosity, 36.7% were tested when they were young children, by their parents, 17.4% were tested at the doctor’s request and 8.7% of them were tested as a school enrolment requirement. The level of SCD knowledge of the students did not significantly differ based on their socio-demographic characteristics (Table 5).

**TABLE 4:** Respondents’ sickle cell disease status awareness, genotype and reason for testing.

Variable	Frequency	%
**SCD status known (*n* = 270)**		
Yes	196	72.6
No	74	27.4
**Genotype (*n* = 196)**		
AA	155	79.1
AS	37	18.9
SS	4	2.0
**Reason genotype testing was done (*n* = 196)**		
I just wanted to know.	73	37.2
It was done for me as a child.	72	36.7
Doctor’s request	34	17.4
It was needed for admission into the school.	17	8.7

SCD, sickle cell disease; AA, haemoglobin AA (normal haemoglobin genotype); AS, haemoglobin AS (sickle cell trait); SS, haemoglobin SS (sickle cell disease).

**TABLE 5:** Factors associated with knowledge of sickle cell disease.

Variable	Knowledge						*X* ^2^	*p*
	Good (*n* = 170)		Poor (*n* = 100)		Total (*n* = 270)			
	*n*	%	*n*	%	*n*	%		
**Age group**							**4.50**	**0.095**
10–13	17	85.0	3	15.0	20	100.0	-	-
14–17	147	61.3	93	38.7	240	100.0	-	-
18–21	6	60.0	4	40.0	10	100.0	-	-
**Gender**							**0.03**	**0.862**
Female	107	62.6	64	37.4	171	100.0	-	-
Male	63	63.6	36	36.4	99	100.0	-	-
**Religion**							**0.05**	**0.830**
Christianity	89	63.6	51	36.4	140	100.0	-	-
Islam	81	62.3	49	37.7	130	100.0	-	-
**Ethnicity**							**0.59**	**0.745**
Hausa	14	60.9	9	39.1	23	100.0	-	-
Igbo	39	67.2	19	32.8	58	100.0	-	-
Yoruba	117	61.9	72	38.1	189	100.0	-	-
**Mother’s highest educational level**							**3.00**	0.374*
No formal education	5	55.6	4	44.4	9	100.0	-	-
Primary	25	55.6	20	44.4	45	100.0	-	-
Secondary	73	61.3	46	38.7	119	100.0	-	-
Tertiary	67	69.1	30	30.9	97	100.0	-	-
**Father’s highest educational level**							**2.55**	0.438*
No formal education	2	50.0	2	50.0	4	100.0	-	-
Primary	24	63.2	14	36.8	38	100.0	-	-
Secondary	49	57.0	37	43.0	86	100.0	-	-
Tertiary	95	66.9	47	33.1	142	100.0	-	-
**Mother’s employment status**							**2.18**	**0.140**
Employed	94	67.1	46	32.9	140	100.0	-	-
Unemployed	76	58.5	54	41.5	130	100.0	-	-
**Father’s employment status**							**3.68**	**0.055**
Employed	132	66.3	67	33.7	199	100.0	-	-
Unemployed	38	53.5	33	46.5	71	100.0	-	-

* , Fisher’s exact *p*-value.

Knowing their SCD status was significantly associated with their attitude towards the disease (*p* = 0.014), and a larger proportion of those who knew their SCD status had a positive attitude. However, their reported genotype did not significantly influence their knowledge or attitude regarding SCD (Table 6).

**TABLE 6:** Sickle cell disease knowledge and attitude associated with sickle cell status.

Variable	Knowledge								Attitude							
	Good		Poor		Total		*X* ^2^	*p*	Positive		Negative		Total		*X* ^2^	*p*
	*n*	%	*n*	%	*n*	%			*n*	%	*n*	%	*n*	%		
**SCD status known (*n* = 270)**							0.93	0.336								0.0145*
Yes	120	61.2	76	38.8	196	100.0	-	-	193	98.5	3	1.5	196	100.0	-	-
No	50	67.6	24	32.4	74	100.0	-	-	68	91.9	6	8.1	74	100.0	-	-
**Total**	170	63.0	100	37.0	270	100.0	-	-	261	96.7	9	3.3	270	100.0	-	-
**Genotype (*n* = 196)**																
AA	91	58.7	64	41.3	155	100.0	-	-	152	98.1	3	1.9	155	100.0	-	-
AS	28	75.7	9	24.3	37	100.0	-	-	37	100.0	0	0.0	37	100.0	-	-
SS	1	25.0	3	75.0	4	100.0	-	-	4	100.0	0	0.0	4	100.0	-	-
Total	120	61.2	76	38.8	196	100.0	-	0.151*	193	98.5	3	1.5	196	100.0	-	1.000*

SCD, sickle cell disease.

* , Fisher’s exact *p*-value.

## Discussion

In this study, the level of SCD awareness was high, about two-thirds had a good level of knowledge of SCD, and over 95.0% had a positive attitude towards the disease. Over 70.0% of the respondents were aware of their genotypes, whilst the SCD prevalence was 2.0%. The mean age of the respondents was 15.2 ± 1.3 years, the female-to-male ratio was about 2:1 and the highest levels of education of the respondents’ mothers and fathers, an important factor in the awareness and prevention of SCD, were mostly secondary school and higher education, respectively, similar to the findings of a study carried out amongst secondary school students in Federal Capital Territory (FCT), Abuja, Nigeria,^12^ where the respondents’ mean age was 15.2 ± 2.1 years and a large number of the respondents’ fathers (80.7%) and mothers (70.2%), respectively, had at least secondary school education. However, it is contrary to a study performed amongst secondary school students in Jos, Nigeria, where the respondents’ ages ranged from 9 to 25 years with a mean age of 17 ± 3 years and there were more males (51.0%) than females (49.0%).^10^ The male preponderance of the students in the Jos study could be because of the cultural tendency to educate mostly male children in Northern Nigeria.

According to this study, the majority of the respondents knew about SCD, whilst the remaining one-tenth did not know about SCD. This compares well with a study carried out amongst secondary school students in Jos which found that 113 (97.4%) of respondents were aware of SCD, whilst only three (2.6%) of them were not aware.^10^ The difference in the percentage of those who were aware of SCD may be accounted for by the huge difference in sample size (300–137), the geographical locations involved, amongst other factors. As found in this study, the major sources of information about SCD include health professionals, the internet, friends/family, television/radio and posters. The study in Jos corroborated this finding reporting that 50 respondents (36.5%) got information about SCD from health professionals, 16 respondents (11.1%) from the Internet, 19 respondents (13.8%) from friends and 25 respondents (18.2%) from family,^10^ whereas in another study carried out amongst 329 undergraduate students of a tertiary institution in Abakaliki, Ebonyi State, Nigeria, 115 respondents (35.0%) reported that lectures were the most frequent source of information about SCD, 63 respondents (19.1%) named health workers, 55 respondents (16.7%) named friends and colleagues, 37 respondents (11.2%) named family members, 34 respondents (10.3%) credited the radio and television, 11 respondents (3.3%) named the library, five respondents (1.5%) credited the internet, whilst one respondent (0.3%) named posters as their source of information.^18^ These differences may be because of factors such as the preference of different modes of learning in each region, the age and educational levels in the Abakaliki study, social exposure, a more civil environment and the prevalence for different teaching and learning techniques in each region.

The majority of the respondents in this study chose testing before marriage as a method of preventing SCD, followed by genetic counselling. This can be compared to a study in Kampala, Uganda where testing before marriage (40.2%) and genetic counselling (21.6%) were chosen as methods of preventing SCD, whereas in the same study, one third of the students (38.2%) did not know of any methods of SCD prevention.^14^ This may be because of the frequency and the quality of the information available about SCD in Lagos, Nigeria compared to Uganda, given that SCD is more prevalent in Nigeria, where the highest burden of the disease in the African region is found.^17^

Similar to the current study, a study performed in Ghana found that a majority of the students had a strong belief that SCD is not an evil disease (70.6%) but an inherited disease acquired from one’s parents (48.3%).^13^ Also to corroborate these findings, a study in Jos also reported that a quarter of the students (25.5%) had the wrong belief that SCD is caused by evil spirits.^10^ In another study, though amongst nursing students in Sokoto, Nigeria, the majority (96.4%) of them considered the disease as being beyond spiritual, a serious disease that was potentially fatal (45.0%).^19^ This is an unexpected similarity because the students in Lagos were not specifically receiving any medical training.

Out of the total number of respondents who were aware of SCD, about two-thirds had good knowledge of the disease, lower than those found in a study performed amongst youths in Yaba, Lagos, Nigeria which showed that the majority (80.0%) had good knowledge of SCD.

This higher level of knowledge may be because Yaba, Lagos, Nigeria is surrounded by many health facilities and higher institutions of learning and so, the respondents are more likely to have a good knowledge of SCD.^20^ Also, in the study done in Yaba, the respondents were older, which may suggest that there is a direct relationship between higher age and good knowledge of SCD. In comparison with the current study, a lower proportion (65%) of students with a positive attitude towards SCD was found in a similar study in Osogbo in Osun State Nigeria.^21^ This might be explained by the metropolitan nature of Lagos compared to Osogbo.

In this study, about three-quarters knew their SCD status, meaning that they reported that they did or did not have SCD, whilst about a quarter of them did not know their genotype. The majority of the respondents who knew their genotype (79.1%) were of genotype AA, 18.9% were of genotype AS and 2.0% were of genotype SS. This can be compared to a study in Jos where in terms of knowing their SCD status, 89.6% believed in the importance of knowing one’s genotype, whilst only 59.2% knew their genotype. Amongst those who claimed to know their genotype, 59.2% were of genotype AA, 11.1% were of genotype AS, whilst 29.6% had other combinations.^10^ These differences in the two studies may be because of the disparity in the levels of literacy and social exposure between the western and northern parts of the country amongst other factors like social status and availability of testing.

Whilst the current study found no factors associated with the knowledge of SCD and attitude to be influenced only by knowing one’s SCD status, other studies found SCD knowledge to be associated with the area of study, religion and age (each *p* < 0.001),^22^ higher level of education (*p* = 0.030) and knowing a relative with SCD (*p* = 0.010),^13^ and attitude to be predicated on religion, knowledge of SCD and marital status, each *p* < 0.001,^22^ though the students in these studies were undergraduates and were likely to be older.

### Strengths and limitations of the study

This was a school-based study; therefore, the students were able to provide their responses without parental pressure that may have existed at home; however, the self-reported genotype of the respondents was, therefore, unconfirmed.

## Conclusion and recommendation

The majority of the respondents had good knowledge of SCD, but this did not translate into a mostly positive attitude. However, a gap in knowledge regarding the genetic nature and inheritance pattern of the disease still exists. The majority of the respondents were not knowledgeable about the prevention of SCD considering that they are at the point in life just preceding the decision to bear children. To adequately prepare our young people to prevent SCD routine screening, counselling towards attitudinal change and teachings inclusive of the inheritance pattern of SCD should be part of the secondary school curriculum in Nigeria.
